# *RET* rearrangements are relevant to histopathologic subtypes and clinicopathological features in Thai papillary thyroid carcinoma patients

**DOI:** 10.3389/pore.2023.1611138

**Published:** 2023-04-28

**Authors:** Thitima Khonrak, Sasithorn Watcharadetwittaya, Yaovalux Chamgramol, Piyapharom Intarawichian, Raksawan Deenonpoe

**Affiliations:** ^1^ Department of Pathology, Faculty of Medicine, Khon Kaen University, Khon Kaen, Thailand; ^2^ Cholangiocarcinoma Research Institute (CARI), Khon Kaen University, Khon Kaen, Thailand

**Keywords:** papillary thyroid carcinoma, RET rearrangements, *CCDC6*::*RET* rearrangement, *NCOA4*::*RET* rearrangement, gene rearrangements

## Abstract

**Background:** Papillary thyroid carcinoma (PTC) is the most common type of thyroid cancer*.* The *RET* gene rearrangements *CCDC6*::*RET* and *NCOA4*::*RET* are the most common *RET* gene rearrangements in PTC patients. Different *RET*::*PTC* rearrangements are associated with different PTC phenotypes.

**Methods:** Eighty-three formalin-fixed paraffin-embedded (FFPE) PTC samples were examined. The prevalence and expression levels of *CCDC6*::*RET* and *NCOA4*::*RET* were determined using semi-quantitative polymerase chain reaction (qRT-PCR). The association of these rearrangements with clinicopathological data was investigated.

**Results:** The presence of *CCDC6*::*RET* rearrangement was significantly associated with the classic subtype and absence of angio/lymphatic invasion (*p* < 0.05). While *NCOA4*::*RET* was associated with the tall-cell subtype, and presence of angio/lymphatic invasion and lymph node metastasis (*p* < 0.05). Multivariate analysis demonstrated that an absence of extrathyroidal extension and extranodal extension were independent predictive factors for *CCDC6*::*RET*, whereas the tall-cell subtype, large tumor size, angioinvasion, lymphatic invasion and perineural invasion were independent predictive factors for *NCOA4*::*RET* (*p* < 0.05). However, the mRNA expression level of *CCDC6*::*RET* and of *NCOA4*::*RET* were not significantly associated with clinicopathological data.

**Conclusion:** *CCDC6*::*RET* was correlated with an innocent PTC subtype and characteristics, but *NCOA4*::*RET* correlated with an aggressive phenotype of PTC. Therefore, these *RET* rearrangements strongly associated with clinicopathological phenotypes and can be used as predictive markers in PTC patients.

## Introduction

Papillary thyroid carcinoma (PTC) is the most common form of well-differentiated thyroid cancer, originating from thyroid follicular cells. PTC is also the most prevalent form of thyroid cancer overall, contributing to 75%–85% of thyroid-cancer cases (1). Although its incidence is rapidly increasing, PTC has the best overall prognosis of any type of thyroid cancer.

*BRAF* and *RAS* genes, as well as rearrangements of the *RET* gene are strongly associated with PTC, respectively. These mutations are associated with mitogen-activated protein kinase (MAPK) and phosphatidylinositol-3 kinase (PI3K)/AKT signaling pathways that affect proliferation and differentiation in cancer cells. *RET* fusions/rearrangements, are the most frequent molecular alterations reported in PTC, being present in about 10%–40% of cases worldwide, but in 45%–60% of cases involving adolescents and children with sporadic PTC (2–5). The major fusion partner genes in such rearrangements are *CCDC6* and *NCOA4* (also known as *ELE1*). These two fusions account for approximately 90% of such cases (6).

The *RET* (rearranged during transfection) gene is a proto-oncogene located on the long arm of chromosome 10 (10q11.2), and consists of three parts; the extracellular segment, transmembrane segment and an intracellular segment (7); the intracellular segment is an important part that causing of *RET*::*PTC* rearrangements. The fusion of tyrosine kinase domain with the 5′region of another gene such as *CCDC6* and *ELE1,* generates chimeric products collectively named *RET*::*PTCs*. These result in ligand-independent dimerization and constitutive activation of these chimeric proteins triggers *RAS* which activates *RAF* kinases, and the downstream signaling cascade, causing proliferation and differentiation in cancer cells.

*CCDC6*::*RET* is formed by fusion with the *CCDC6* (also designated *H4*) gene and *NCOA4*::*RET* by fusion with the *NCOA4* (also designated *ELE1*, *RFG* or *ARA70*). These rearrangements are associated with papillary thyroid carcinoma cases with different characteristics; *CCDC6*::*RET* is more associated with sporadic thyroid cancer (8, 9) and with older patients and classic subtypes of papillary carcinoma (8–10). The *NCOA4*::*RET* rearrangement correlates with a more aggressive phenotype and a more advanced stage of PTC. Those fusion genes are also strongly associated with short radiation latency and solid subtypes of PTC (8, 9, 11–13). *RET*::*PTC*-mediated signaling depends on a specific *RET* tyrosine residue, Y1062 (numbering refers to the full-length RET protein). Both types of *RET* fusion have comparable levels of Y1062 phosphorylation but, MAPK phosphorylation is higher in *NCOA4*::*RET* than in *CCDC6*::*RET* (14). Therefore, the two rearrangements have the same enzymatic activity and autophosphorylation levels, but activation of MAPK is greater by *NCOA4*::*RET* than *CCDC6*::*RET*.

Although the global prevalence of major *RET*::*PTC* fusions in PTC patients has been reported, but no data are available specifically for Thailand. Thus, this study aimed to determine the prevalence of *CCDC6*::*RET* and *NCOA4*::*RET* fusions in Thai papillary thyroid carcinoma patients. Prevalence and expression levels of *CCDC6*::*RET* and *NCOA4*::*RET* rearrangements in PTC patients were determined using semi-quantitative polymerase chain reaction (qRT-PCR). The association of these *RET*::*PTC* rearrangements with clinicopathological data was also investigated to test the hypothesis that *CCDC6*::*RET* and *NCOA4*::*RET* fusion genes are differently associated with aggressive characteristics in PTC.

## Material and methods

### Study population

The study was approved by the Ethics Committee for Human Research, Khon Kaen University (HE641011). Formal written informed consent was not required with a waiver by the appropriate IRB and/or national research ethics committee. Formalin-fixed paraffin-embedded (FFPE) tissue of patients (*n* = 83) who had been diagnosed with papillary thyroid carcinoma by pathologists at Pathology services, Department of Pathology in the years 2010–2014 were investigated. All cases were included for this study by clinical data from medical records at Srinagarind hospital, Khon Kaen, Thailand, including sex, age, clinicopathological information including size of tumor, subtype of PTC, angioinvasion, lymphatic invasion, perineural invasion, extrathyroidal extension, extranodal extension and lymph node metastasis. Data relating to lymph node metastasis and extranodal extension was missing for some patients. *BRAF* and *KRAS* mutations were not included in clinicopathological data according to genetic alterations were not performed in our routine service. As only morphologic features were investigated for pathological diagnosis.

### FFPE slides preparation

Section of formalin-fixed, paraffin-embedded (FFPE) tissues from normal thyroid (*n* = 10), benign multinodular goiter (*n* = 10), PTC (*n* = 83) were stained with hematoxylin and eosin (H&E) and reviewed by two pathologists (SW & PI) to confirm the diagnoses and subclassify the subtypes of papillary carcinomas (Figure 2A). Paraffin blocks of each tumor were examined by pathologists to identify the extent of tumorous tissue. These were then used for RNA extraction.

### RNA extraction and cDNA synthesis

FFPE blocks of all PTC tissues, as well as, benign and normal thyroid, were cut to provide sections of approximately 10 µm in thickness. Ten ribbons from each sample were used for RNA extraction. Tissue deparaffinization was performed using xylene and ethanol (SAV Liquid Production GmbH, Flintsbach am Inn, Germany). RNA was extracted using TRIzol^®^ Reagent (Thermofisher Waltham, Massachusetts, United States) with the addition of 0.2 mL of chloroform per 1 mL of TRIZOL reagent was added. Tissue sections were homogenized in 1 mL of TRIZOL reagent per 50–100 mg of tissue (1 ribbon with 10 µm ≈ 1 mg). Isopropanol per 1 mL TRIZOL reagent was used for precipitating the RNA. The concentration and purity of extracted RNA and protein were measured using a nanodrop spectrophotometer at wavelengths of 260 nm (A260) and 280 nm (A280), respectively. The concentration of RNA 50 μg/mL absorbed light at a wavelength of 260 nm in general. Complementary deoxyribonucleic acid (cDNA) was synthesized from messenger RNA using reverse transcription PCR and the RevertAid First Strand cDNA Synthesis Kit (Thermofisher Waltham, Massachusetts, United States) following the manufacturer’s instructions. For oligo (dT) 18 or gene-specific primed cDNA synthesis, incubated for 60 min at 42°C. For random hexamer primed synthesis, incubated for 5 min at 25°C followed by 60 min at 42°C. Terminated the reaction by heating at 70°C for 5 min. The reverse transcription reaction product could be directly used in PCR applications or stored at −20°C for less than 1 week.

### Semi-quantitative polymerase chain reaction (qRT-PCR)

Two specific primers were designed based on the sequences deposited in GenBank for *CCDC6*::*RET* and *NCOA4*::*RET* (Table 1). The nucleotide positions of those primers were synthesized as positive control. cDNA (50 ng/μL) and 23 μL of RT-PCR master mix [SYBR Green master mix qPCR kit (Thermo Scientific Ltd., United Kingdom)] were used for the qRT-PCR. The reactions were carried out in triplicate. GAPDH mRNA was used as an internal control and a no-template control (NTC) was included each time. The amplification of cDNA was performed using a Thermal kit (Thermo Scientific Ltd., United Kingdom) with the following 40 cycles of amplification conditions in Supplementary Table S1. The cuff off cycle threshold (Ct) value was 37th cycle. Cases with Ct values ≤37 were categorized as positive for the specific rearrangement. Post-PCR fluorescence melting curve analysis was done to ensure that only a single product had been amplified. The CT values of *CCDC6*::*RET* and *NCOA4*::*RET* rearrangements were calculated, and data were expressed as the fold change of control (normal) using 2^-(ΔΔ Ct)^.

**TABLE 1:** The primer sequences.

Name	Primers	Product size (bp)
GAPDH	F-GTCTCCTCTGACTTCAACAGCG	97
	R-ACCACCCTGTTGCTGTAGCCAA	
*CCDC6*::*RET*	F-ATACTGGAGAACAGGCCATCTG	207
	R-CGTTGCCTTGACCACTTTTCC	
*NCOA4*::*RET*	F-CCTTTCAGCGAATGGCTCCT	136
	R- ATT​CCC​ACT​TTG​GAT​CCT​CCT	

Nucleotide positions of the primers are based on the sequences deposited in GenBank: *CCDC6*::*RET* (accession no.AF395885.1) and *NCOA4*::*RET* (accession no. S71225.1).

### Gel electrophoresis and sequencing

PCR products from the cases positive according to qRT-PCR were analyzed by electrophoresis through a 2% gel electrophoresis and visualized by ethidium bromide staining. The anticipated sizes of *CCDC6*::*RET* and *NCOA4*::*RET* products were 207 and 136 bp respectively. These bands were interpreted by visualization with a gel documentation system. Barcode-Tagged Sequencing (BTSeq ™) (U2Bio, Korea) was also applied for positive cases to obtain sequences of the PCR products.

### Statistical analyses

Distributions of all continuous variables were tested for normality using the Shapiro-Wilk test. None was found to be normally distributed, requiring the use of non-parametric tests for further analysis. Comparison of categorical variables was carried out using Chi-square tests. Kruskal-Wallis tests and Mann-Whitney U tests to determine whether medians were different between comparison groups and to test for different expression levels between normal, benign goiter and PTC groups. Simple logistic regression and multiple logistic regression were used for backward stepwise univariate and multivariate analyses to identify independent prognostic factors for PTC patients using categorical data. Simple linear regression and multiple linear regression were used for continuous data. Variable factors with p value equal or less than 0.25 from univariate analysis were included to multivariate analysis to investigate the correlation between RET rearrangement and clinical characteristics.

The Ct values in the qRT-PCR assay of *CCDC6*::*RET* and *NCOA4*::*RET* levels were calculated, and data were expressed as the fold-change relative to controls using 2(^−ΔΔCt^). Results were regarded as statistically significant at *p*-values lower than 0.05. The statistical analyses were done using IBM SPSS statistics software version 28.0 (IBM, Chicago, IL, United States).

## Results

### Prevalence of *RET* rearrangements

**FIGURE 1:**
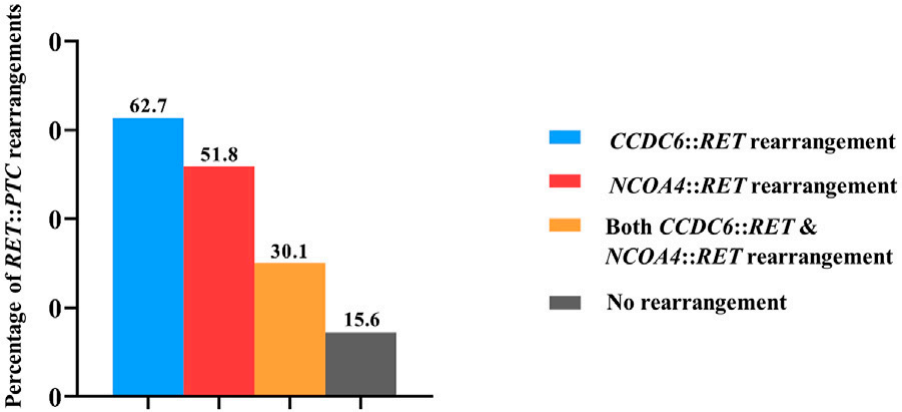
The frequency of *RET* rearrangements in PTC patients. In detail, *CCDC6*::*RET* rearrangements were detected in 62.7% (52/83), *NCOA4*::*RET* rearrangements in 51.8% (43/83), both *CCDC6*::*RET* and *NCOA4*::*RET* rearrangements co-occurred in 30.1% (25/83) and 15.6% (13/83) PTCs were negative for both *RET*::*PTC* rearrangements.

Eighty-three PTC patients included 59 females and 24 males with an average age of 48.21 years (range 17–79 years). Results from qRT-PCR showed the absence of *CCDC6*::*RET* or *NCOA4*::*RET* rearrangements in normal tissues (*n* = 10) and benign (multinodular goiter, *n* = 10) cases. On the other hand*, CCDC6*::*RET* rearrangements were detected in 52 PTC cases (62.7%), and *NCOA4*::*RET* rearrangements in 43 cases (51.8%). Both *CCDC6*::*RET* and *NCOA4*::*RET* rearrangements co-occurred in 25 PTC tissues (30.1%). Thirteen PTCs (15.6%) tested negative for either *RET*::*PTC* rearrangement (Figure 1).

### The association between *RET* rearrangements and clinicopathological data in PTC patients

**FIGURE 2:**
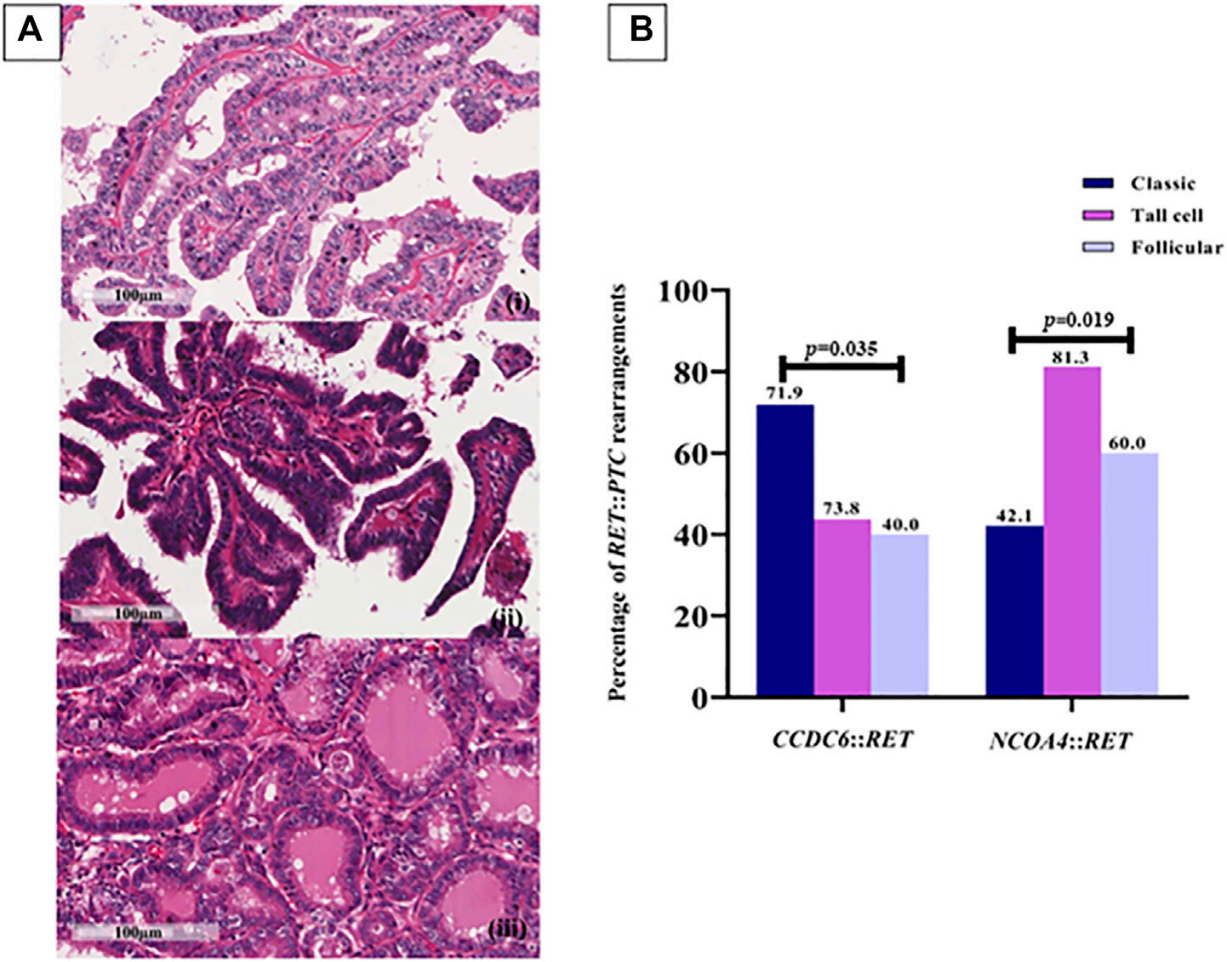
*RET* rearrangements in each PTC subtype. **(A)** Papillary thyroid carcinoma subtypes. (i) Classic PTC; consists of papillae with fibrovascular core line by neoplastic cell characterized by increased nuclear size, nuclear overlapping, nuclear features like clear, ground glass appearance and oval shape (400X, scale bar = 100 µm); (ii) Tall cell PTC; abundant eosinophilic cytoplasm and nuclear features characteristic of classic subtype (400X, scale bar = 100 µm); (iii) Follicular PTC; follicular architecture but enlarged nuclei with cytologic features of PTC (400X, scale bar = 100 µm). **(B)** The prevalence of *RET*::*PTC* rearrangements in each PTC subtype; 41 (71.9%) and 24 (42.1%) cases of the classic subtype are positive for *CCDC6*::*RET* and *NCOA4*::*RET*, respectively, 7 (43.8%) cases of the tall-cell subtype were positive for *CCDC6*::*RET* and 13 (81.3%) cases for *NCOA4*::*RET* rearrangement, 4 (40%) cases of follicular PTC for *CCDC6*::*RET* and 6 (60%) cases for *NCOA4*::*RET*. Either the frequency of *CCDC6*::*RET* or *NCOA4*::*RET* rearrangements are significantly different in those subtypes (*p* = 0.035, *p* = 0.019).

PTCs have been categorized into classic, tall-cell subtypes and follicular variants. A correlation between the histological subtype and the type of *RET* rearrangement was observed; 71.93% of the classic subtype samples were positive for *CCDC6*::*RET* and 42.11% for *NCOA4*::*RET*, 43.75% of the tall-cell subtype tissues were positive for *CCDC6*::*RET* and 81.25% of them were *NCOA4*::*RET* positive. Both the frequency of *CCDC6*::*RET* and NCOA4::RET rearrangements were significantly different in those subtypes (OR = 0.27, 95% CI = 0.11–0.71) (Figure 2; Table 2)*.*

**TABLE 2:** Correlation between *RET* rearrangements and clinicopathological data using Chi-square analysis.

Variable	Category	N	*CCDC6*::*RET* rearrangement (%)	*p*	*NCOA4*::*RET* rearrangement (%)	*p*	*CCDC6*::*RET* and *NCOA4*::*RET* rearrangement (%)	*p*
Gender	Male	24	15 (62.5)	0.986	14 (58.3)	0.448	9 (37.5)	0.350
	Female	59	37 (62.7)		29 (49.2)		16 (27.1)	
Age	<55	53	39 (73.6)	**0.006**	25 (47.2)	0.261	17 (32.1)	0.606
	≥55	30	13 (43.3)		18 (60.0)		8 (26.7)	
Tumor size	≤1	7	5 (71.4)	0.311	4 (57.1)	0.501	3 (42.9)	0.746
	1 < T ≤ 2	18	13 (72.2)		8 (44.4)		5 (27.8)	
	2 < T ≤ 4	40	26 (65.0)		19 (47.5)		13 (32.5)	
	>4	18	8 (44.4)		12 (66.7)		4 (22.2)	
PTC subtype	Classic	57	41 (71.9)	**0.035**	24 (42.1)	**0.019**	14 (24.6)	0.258
	Tall cell	16	7 (43.8)		13 (81.3)		7 (43.8)	
	Follicular^a^	10	4 (40.0)		6 (60.0)		4 (40.0)	
Angioinvasion	Absent	43	33 (76.7)	**0.006**	15 (34.9)	**0.001**	13 (30.2)	0.982
	Present	40	19 (47.5)		28 (70.0)		12 (30.0)	
Lymphatic invasion	Absent	46	37 (80.4)	**<0.001**	17 (37.0)	**0.003**	16 (34.8)	0.343
	Present	37	15 (40.5)		26 (70.3)		9 (24.3)	
Perineural invasion	Absent	69	46 (66.7)	0.093	33 (47.8)	0.107	22 (31.9)	0.437
	Present	14	6 (42.9)		10 (71.4)		3 (21.4)	
Extrathyroidal extension	Absent	67	48 (71.6)	**<0.001**	29 (43.3)	**0.001**	21 (31.3)	0.619
	Present	16	4 (25.0)		14 (87.5)		4 (25.0)	
Lymph node metastasis^b^	Absent	12	9 (75.0)	0.129	4 (33.3)	**<0.001**	4 (33.3)	0.425
	Present	39	20 (51.3)		29 (74.4)		14 (35.9)	
Extranodal extension^b^	Absent	31	22 (71.0)	**0.030**	18 (58.1)	**0.013**	13 (41.9)	0.180
	Present	19	7 (36.8)		14 (73.7)		5 (26.3)	

^a^ The follicular variant (*n* = 10) divided into 5 encapsulated FVPTC and 5 infiltrative FVPTC; 2 encapsulated FVPTC and 2 infiltrative FVPTC presented *CCDC6*::*RET*, 1 encapsulated FVPTC and 3 infiltrative FVPTC presented *NCOA4*::*RET*.

FVPTC, follicular variant of papillary thyroid carcinoma.

^b^ Some missing data.

Bold values mean “statistical significant”.

Categorical variables were compared using Chi-square tests. Neither type of rearrangement was statistically significantly associated with gender. The *CCDC6*::*RET* rearrangement was significantly associated with patients younger than 55 years of age (*p* = 0.006) and classic subtype (*p* = 0.035). For clinicopathological markers, the *CCDC6*::*RET* rearrangement was significantly associated with an absence of angioinvasion (*p* = 0.006) and of lymphatic invasion extrathyroidal extension and extranodal extension (*p*= <0.001 and 0.030). *NCOA4*::*RET* rearrangements were statistically significantly associated with the tall cell subtype (*p* = 0.019) and angioinvasion (*p* = 0.001), lymphatic invasion (*p* = 0.003), lymph node metastasis (*p*= <0.001), extrathyroidal extension (*p* = 0.001) and extranodal extension (*p* = 0.013). Co-occurrence of *CCDC6*::*RET* and *NCOA4*::*RET* rearrangements was not significantly associated with clinicopathological data in PTC patients (Table 2).

**FIGURE 3:**
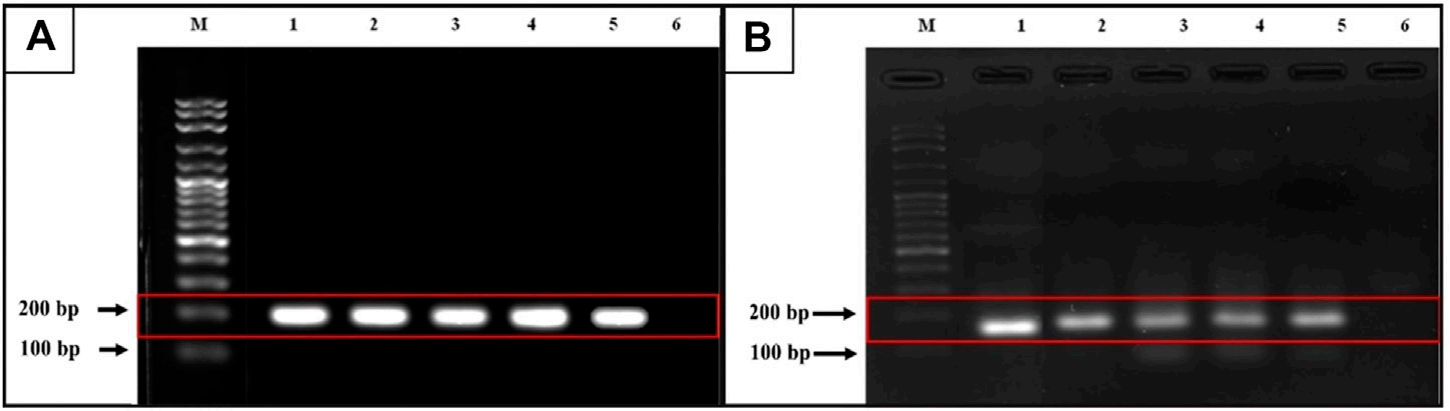
Gel electrophoresis from qRT-PCR products. **(A)** *CCDC6*::*RET* rearrangement (expected band size 207 bp). M = 100 bp ladder, lane 1 is a positive control, lanes 2 and 3 = classic subtype, lane 4 and lane 5 = follicular variant, lane 6 = undetectable case **(B)** *NCOA4*::*RET* rearrangement (expected band size 136 bp). M = 100 bp ladder, lane 1 is a positive control, lane 2 = classic subtype, lane 3 and 4 = tall-cell, lane 5 = follicular variant and lane 6 = undetectable case.

**FIGURE 4:**
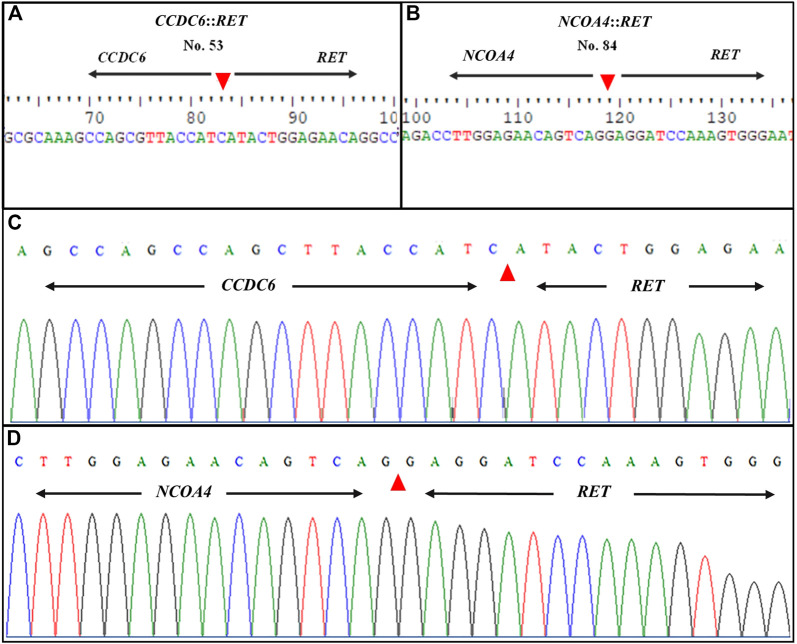
Representative sequence lengths of qRT-PCR products and electropherograms of *RET* rearrangements. **(A)** *CCDC6*::*RET*; *RET* fusion with the *CCDC6* gene and **(B)** *NCOA4*::*RET; RET* fusion with the *NCOA4* gene **(C)** Showing formed, distinctive single-coloured peaks of DNA electropherogram of *CCDC6*::*RET* and **(D)** *NCOA4*::*RET* rearrangements. The patient’s identification number is listed at the top of each panel. Nucleotide sequences around the fusion points of the two different types of *RET* rearrangement transcripts are shown. Arrowheads indicate the fusion point.

Following gel electrophoresis and visualization, aliquots of each qRT-PCR product were sent to BTSeq ™ (U2Bio, Korea) for sequencing. The *CCDC6*::*RET* and *NCOA4*::*RET* products were 207 and 136 bp in length, respectively, as expected (Figure 3). The sequencing results showing the fusion points and sequencing electropherograms are shown in Figure 4.

### Clinicopathological variables as predictive factors for *RET* rearrangements

Clinicopathological parameters of the 83 PTC patients were analyzed as dependent and independent predictive factors using simple logistic regression and multiple logistic regression with 95% CI values. Being younger than 55 years was a dependent predictive factor for the *CCDC6*::*RET* rearrangement (OR = 0.27, 95% CI = 0.11–0.71; *p* = 0.007). The absence of angioinvasion (OR = 0.27, 95% CI = 0.11–0.70; *p* = 0.007), absence of lymphatic invasion (OR = 0.17, 95% CI = 0.06–0.44; *p* < 0.001), of extrathyroidal extension (OR = 0.13, 95% CI = 0.04–0.46; *p* = 0.001) and of extranodal extension (OR = 0.24, 95% CI = 0.07–0.80; *p* = 0.021) were dependent predictive factors for *CCDC6*::*RET*. In multivariate analysis, controlling for the influence of other factors, an absence of extrathyroidal extension (OR = 0.25, 95% CI = 0.02–0.84, *p* = 0.032) and of extranodal extension (OR = 0.16, 95% CI = 0.03–0.80, *p* = 0.026) were significant independent predictive factors for *CCDC6*::*RET* (Table 3).

**TABLE 3:** Univariate and multivariate analysis of the clinicopathological factors with *RET* rearrangements by the simple logistic regression and multiple logistic regression.

Variable	Category	n (%)	*CCDC6*::*RET* rearrangement				n (%)	*NCOA4*::*RET* rearrangement			
			Univariate analysis		Multivariate analysis			Univariate analysis		Multivariate analysis	
			OR (95% CI)	*p*	OR (95% CI)	*p*		OR (95% CI)	*p*	OR (95% CI)	*p*
Gender	Male	15 (62.5)	1	0.986			14 (58.3)	1	0.449		
	Female	37 (62.7)	1.01 (0.38–2.69)				29 (49.2)	0.69 (0.26–1.80)			
Age	<55	39 (73.6)	1	**0.007**	1	0.159	25 (47.2)	1	0.263		
	≥55	13 (43.3)	0.27 (0.11–0.71)		0.27 (0.04–1.67)		18 (60.0)	1.68 (0.68–4.17)			
Tumor size	≤1	5 (71.4)	1		1	0.056	4 (57.1)	1		1	
	1 < T ≤ 2	13 (72.2)	1.04 (0.15–7.22)	0.968			8 (44.4)	0.60 (0.10–3.49)	0.570		
	2 < T ≤ 4	26 (65.0)	0.74 (0.13–4.33)	0.741			19 (47.5)	1.68 (0.13–3.43)	0.064	1.02 (0.00–1.53)	**0.020**
	>4	8 (44.4)	0.32 (0.05–2.11)	0.236	18.3 (0.93–361.64)		12 (66.7)	1.50 (0.25–8.98)	0.066	1.02 (0.00–1.87)	**0.042**
PTC subtype	Classic	41 (71.9)	1				24 (43.8)	1		1	
	Tall cell	7 (43.8)	0.30 (0.10–0.95)	**0.041**	0.75 (0.33–2.58)	0.07	13 (81.3)	5.96 (1.53–23.24)	**0.010**	6.06 (2.15–20.49)	**0.032**
	Follicular	4 (40.0)	1.18 (0.06–1.05)	0.058	1.09 (0.9–3.54)	0.121	6 (60.0)	2.06 (0.52–8.12)	0.300	0.03 (0.40–5.70)	0.173
Angioinvasion	Absent	33 (76.7)	1	**0.007**	0.48 (0.01–1.34)	0.086	15 (34.9)	1	**0.002**	1	**0.019**
	Present	19 (47.5)	0.27 (0.11–0.70)				28 (70.0)	4.36 (1.73–10.95)		16.29 (1.57–168.81)	
Lymphatic invasion	Absent	37 (80.4)	1	**<0.001**	1	0.078	17 (37.0)	1	**0.003**	1	**0.015**
	Present	15 (40.5)	0.17 (0.06–0.44)		0.25 (0.05–1.17)		26 (70.3)	4.03 (1.60–10.17)		44.11 (2.11–922.55)	
Perineural invasion	Absent	46 (66.7)	1	0.101	1	0.218	33 (47.8)	1	0.116	1	**0.039**
	Present	6 (42.9)	0.38 (0.12–1.21)		0.35 (0.07–1.86)		10 (71.4)	2.73 (0.78–9.54)		24.64 (1.18–515.96)	
Extrathyroidal extension	Absent	48 (71.6)	1	**0.001**	1	**0.032**	29 (43.3)	1	**0.005**	1	0.109
	Present	4 (25.0)	0.13 (0.04–0.46)		0.25 (0.02–0.84)		14 (87.5)	9.17 (1.93–43.58)		17.99 (0.53–613.89)	
Lymph node metastasis	Absent	9 (75.0)	1	0.157	0.89 (0.09–1.2)	0.123	4 (33.3)	1	**0.004**	1	0.058
	Present	20 (51.3)	0.35 (0.08–1.50)				29 (74.4)	8.7 (1.96–38.65)		9.03 (0.93–88.02)	
Extranodal extension	Absent	22 (71.0)	1	**0.021**	1	**0.026**	18 (58.1)	1	0.268		
	Present	7 (36.8)	0.24 (0.07–0.80)		0.16 (0.03–0.80)		14 (73.7)	2.02 (0.58–7.03)			

Bold values mean “statistical significant”.

Tall-cell PTC (OR = 5.96, 95% CI = 1.53–23.24, *p* = 0.01), angioinvasion (OR = 4.36, 95% CI = 1.73–10.95; *p* = 0.002), lymphatic invasion (OR = 4.03, 95% CI = 1.60–10.17; *p* = 0.003), extrathyroidal extension (OR = 9.17, 95% CI = 1.93–43.58; *p* = 0.005) and lymph node metastasis (OR = 4.03 95% CI = 1.60–10.17; *p* = 0.004) were dependent predictive factors of the *NCOA4*::*RET* rearrangement. In multivariate analysis, the tall-cell subtype (OR = 6.06, 95% CI = 2.15–20.49, *p* = 0.032), tumor size 2 < T ≤ 4 cm and large tumor size >4 cm (OR = 1.02, 95% CI = 0.00–1.53, 0.00–0.87; *p* = 0.020 and 0.042, respectively), perineural invasion (OR = 24.64, 95% CI = 1.18–515.96; *p* = 0.039) lymphatic invasion (OR = 44.11, 95% CI = 2.11–922.55; *p* = 0.015), were significant independent predictive factors of *NCOA4*::*RET* (Table 3). However, co-occurrence of the two major *RET* fusions was not significantly associated with the clinicopathological data (Table 4).

**TABLE 4:** Univariate and multivariate analysis of the clinicopathological factors with both *CCDC6*::*RET* and *NCOA4*::*RET* rearrangements group by the simple logistic regression and multiple logistic.

Variable	Category	n (%)	Univariate analysis		Multivariate analysis	
			OR (95% CI)	*p*	OR (95% CI)	*p*
Gender	Male	9 (37.5)	1	0.352		
	Female	16 (27.1)	0.62 (0.23–1.70)			
Age	<55	17 (32.1)	1	0.606		
	≥55	8 (26.7)	0.77 (0.29–2.08)			
Tumor size	≤1	3 (42.9)	1			
	1 < T ≤ 2	5 (27.8)	0.51 (0.08–3.16)	0.472		
	2 < T ≤ 4	13 (32.5)	0.64 (0.12–3.30)	0.596		
	>4	4 (22.2)	0.38 (0.06–2.46)	0.310		
PTC subtype	Classic	14 (24.6)	1			0.105
	Tall cell	7 (43.8)	2.39 (0.75–7.60)	0.140	1	
	Follicular	4 (40.0)	2.05 (0.50–8.32)	0.316	2.71 (0.81–9.04)	
Angioinvasion	Absent	13 (30.2)	1	0.982		
	Present	12 (30.0)	0.99 (0.39–2.53)			
Lymphatic invasion	Absent	16 (34.8)	1	0.304		
	Present	9 (24.3)	0.60 (0.23–1.58)			
Perineural invasion	Absent	22 (31.9)	1	0.441		
	Present	3 (21.4)	0.58 (0.15–2.30)			
Extrathyroidal extension	Absent	21 (31.3)	1	0.620		
	Present	4 (25.0)	0.73 (0.21–2.53)			
Lymph node metastasis	Absent	4 (33.3)	1	0.871		
	Present	14 (35.9)	1.12 (0.29–4.39)			
Extranodal extension	Absent	13 (41.9)	1	0.268		
	Present	5 (26.3)	0.49 (0.14–1.72)			

### Determination of mRNA expression levels of *RET* rearrangements in thyroid tissues

**FIGURE 5:**
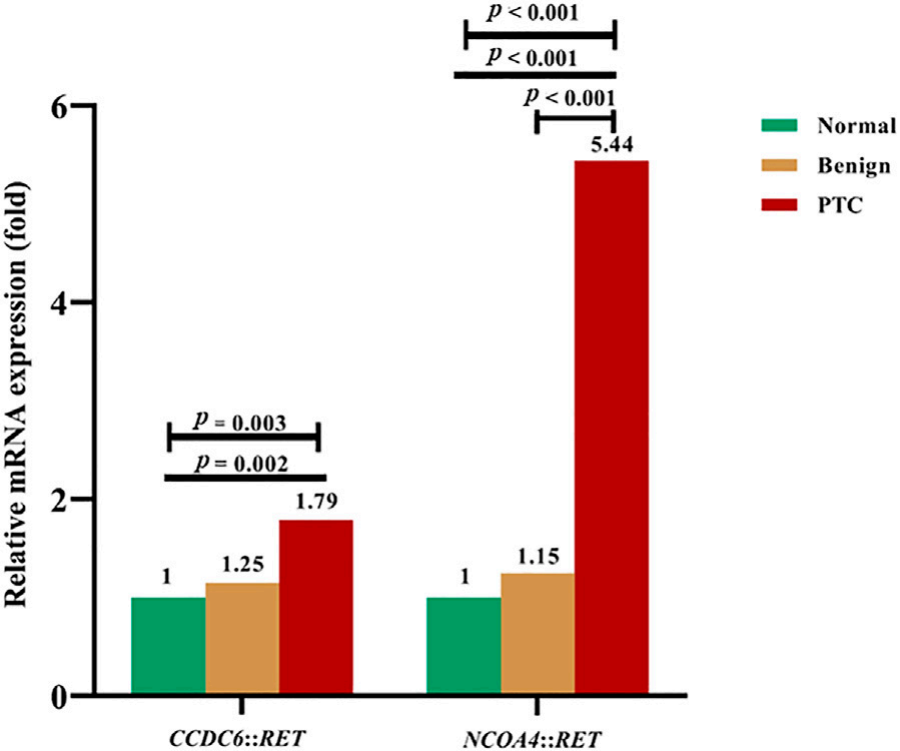
The relative fold-change of mRNA expression levels in *RET* rearrangements transcripts in normal, benign and PTC tissues. For *CCDC6*::*RET*, showing the differentiation of mRNA expression among PTC, benign compared to normal tissue (*p* = 0.003) by Kruskal-Wallis test. Expression of mRNA in PTC is greater than in normal thyroid tissue (*p* = 0.002) by Mann Whitney U test. The *NCOA4*::*RET* mRNA expression, showing the differentiation of mRNA expression among PTC, benign and normal tissues (*p* < 0.001), normal tissue compared to PTC (*p* < 0.001), and benign tissue compared to PTC (*p* < 0.001).

The mRNA expression levels of *CCDC6*::*RET* and *NCOA4*::*RET* rearrangements in PTC compared to benign (multinodular goiter) and normal thyroid tissue are shown in Figure 5. The median mRNA expression levels of *CCDC6*::*RET* transcripts were 0.61 (0.14–1.34), 0.76 (0.36–1.89) and 1.09 (0.20–328.60) in normal, benign and PTC tissues, respectively. The median mRNA expression levels of *NCOA4*::*RET* rearrangement were 0.66 (0.08–2.19), 0.76 (0.36–1.89) and 3.59 (0.69–162.38) in normal, benign and PTC tissues, respectively. The relative *CCDC6*::*RET* mRNA expression levels in PTC (1.79) and benign tissues (1.25) were significantly higher than in normal tissues (*p* = 0.003). The relative mRNA expression levels of *NCOA4*::*RET* transcripts were significantly higher in PTC tissues (5.44) than in benign tissues (1.15) or normal tissues (*p* < 0.001 in both cases) (Figure 5).

### The associations between clinicopathological factors and relative mRNA expression levels of *RET* rearrangements in PTC patients

Associations between clinicopathological factors and mRNA expression levels of *RET* rearrangements were performed using Mann-Whitney U tests and Kruskal-Wallis tests. No statistically significant association with clinicopathological factors was found (Table 5).

**TABLE 5:** Mann-Whitney U tests and Kruskal-Wallis test of the predictive marker with relative *RET* mRNA expression levels in papillary thyroid carcinoma.

Variable	Category	n (%)	*CCDC6*::*RET* mRNA expression		n (%)	*NCOA4*::*RET* mRNA expression	
			Median	*p*		Median	*p*
Gender	Male	15 (62.5)	1.00 (0.20–17.23)	0.762	14 (58.3)	6.13 (1.00–162.38)	0.734
	Female	37 (62.7)	1.12 (0.48–328.60)		29 (49.2)	3.79 (0.69–95.52)	
Age	<55	39 (73.6)	1.31 (0.20–328.60)	0.485	25 (47.2)	3.38 (0.69–95.52)	0.722
	≥55	13 (43.3)	1.06 (0.55–2.63)		18 (60.0)	3.88 (0.97–162.38)	
Tumor size	≤1	5 (71.4)	1.67 (0.49–17.23)	0.100	4 (57.1)	1.92 (0.69–35.59)	0.508
	1 < T ≤ 2	13 (72.2)	1.00 (0.64–328.60)		8 (44.4)	2.94 (0.97–162.38)	
	2 < T ≤ 4	26 (65.0)	1.06 (0.20–61.12)		19 (47.5)	4.88 (0.98–62.24)	
	>4	8 (44.4)	0.90 (0.55–1.94)		12 (66.7)	5.19 (1.32–93.92)	
PTC subtype	Classic	41 (71.9)	1.94 (0.20–328.60)	0.761	24 (42.1)	3.37 (0.69–52.19)	0.294
	Tall cell	7 (43.8)	1.63 (0.64–17.23)		13 (81.3)	6.62 (0.69–162.38)	
	Follicular	4 (40.0)	0.80 (0.49–26.28)		6 (60.0)	3.18 (1.56–52.19)	
Angioinvasion	Absent	33 (76.7)	1.31 (0.53–328.60)	0.091	15 (34.9)	3.79 (0.69–62.24)	0.961
	Present	19 (47.5)	0.87 (0.20–18.48)		28 (70.0)	5.55 (0.69–162.38)	
Lymphatic invasion	Absent	37 (80.4)	1.12 (0.20–328.60)	0.770	17 (37.0)	3.79 (0.97–95.52)	0.377
	Present	15 (40.5)	1.04 (0.48–61.12)		26 (70.3)	2.86 (0.69–162.38)	
Perineural invasion	Absent	46 (66.7)	1.32 (0.20–328.60)	0.057	33 (47.8)	3.38 (0.69–162.38)	0.501
	Present	6 (42.9)	0.66 (0.49–8.07)		10 (71.4)	4.56 (1.06–93.92)	
Extrathyroidal extension	Absent	48 (71.6)	1.03 (0.20–328.60)	0.548	29 (43.3)	2.94 (0.69–95.52)	0.088
	Present	4 (25.0)	1.50 (1.04–2.63)		14 (87.5)	5.55 (1.36–162.38)	
Lymph node metastasis	Absent	9 (75.0)	0.92 (0.70–8.07)	0.814	4 (33.3)	5.39 (0.69–7.37)	0.934
	Present	20 (51.3)	1.18 (0.20–61.12)		29 (74.4)	3.18 (0.69–162.38)	
Extranodal extension	Absent	22 (71.0)	1.02 (0.20–61.12)	0.415	18 (58.1)	3.88 (0.69–93.92)	0.985
	Present	7 (36.8)	0.73 (0.48–4.29)		14 (73.7)	2.86 (0.97–162.38)	

In univariate analysis, the clinicopathological factors were not dependent predictive factors for *CCDC6*::*RET* and *NCOA4*::*RET* mRNA expression levels (Tables 6, 7).

**TABLE 6:** Univariate analysis and multivariate analysis of the clinicopathological factors with relative *CCDC6*::*RET* mRNA expression level by simple linear regression and multiple linear regression.

Variable	Category	n (%)	Univariate analysis		Multivariate analysis	
			Mean diff (95% CI)	*p*	Mean diff (95% CI)	*p*
Gender	Male	15 (62.5)	1	0.416		
	Female	37 (62.7)	11.57 (−16.77–39.92)			
Age	<55	39 (73.6)	1	0.382		
	≥55	13 (43.3)	−13.02 (−42.62–16.60)			
Tumor size	≤1	5 (71.4)	1		1	
	1 < T ≤ 2	13 (72.2)	28.87 (−19.26–76.99)	0.234	−6.90 (−15.49–1.70)	0.111
	2 < T ≤ 4	26 (65.0)	0.37 (−44.29–45.02)	0.987		
	>4	8 (44.4)	−3.12 (−55.26–49.01)	0.905		
PTC subtype	Classic	41 (71.9)	1			
	Tall cell	7 (43.8)	−9.23 (−47.66–29.20)	0.632		
	Follicular	4 (40.0)	−5.68 (−54.91–43.54)	0.817		
Angioinvasion	Absent	33 (76.7)	1	0.295	1	
	Present	19 (47.5)	−13.99 (−40.54–12.57)			
Lymphatic invasion	Absent	37 (80.4)	1	0.659		
	Present	15 (40.5)	−6.30 (−34.78–22.18)			
Perineural invasion	Absent	46 (66.7)	1	0.607		
	Present	6 (42.9)	−10.40 (−50.76–29.96)			
Extrathyroidal extension	Absent	48 (71.6)	1	0.674		
	Present	4 (25.0)	−10.22 (−58.65–38.22)			
Lymph node metastasis	Absent	9 (75.0)	1	0.532		
	Present	20 (51.3)	2.88 (−6.46–12.21)			
Extranodal extension	Absent	22 (71.0)	1	0.515		
	Present	7 (36.8)	−3.24 (−13.33–6.84)			

**TABLE 7:** Univariate analysis and multivariate analysis of the clinicopathological factors with relative *NCOA4*::*RET* mRNA expression level by simple linear regression and multiple linear regression.

Variable	Category	n (%)	Univariate analysis		Multivariate analysis	
			Mean diff (95% CI)	*p*	Mean diff (95% CI)	*p*
Gender	Male	14 (58.3)	1	0.363		
	Female	29 (49.2)	−9.55 (−30.49–11.40)			
Age	<55	25 (47.2)	1	0.356		
	≥55	18 (60.0)	9.10 (−10.59–28.79)			
Tumor size	≤1	4 (57.1)	1			
	1 < T ≤ 2	8 (44.4)	−10.76 (−51.46–29.94)	0.596		
	2 < T ≤ 4	19 (47.5)	0.73 (−35.68–37.13)	0.968		
	>4	12 (66.7)	−3.62 (−41.99–34.76)	0.850		
PTC subtype	Classic	24 (42.1)	1			0.051
	Tall cell	13 (81.3)	21.60 (0.02–43.18)	0.050	1	
	Follicular	6 (60.0)	9.74 (−18.95–38.43)	0.497	20.5 (0.23–40.77)	
Angioinvasion	Absent	15 (34.9)	1	0.374		
	Present	28 (70.0)	9.03 (−11.26–29.32)			
Lymphatic invasion	Absent	17 (37.0)	1	0.808		
	Present	26 (70.3)	2.43 (−17.60–22.45)			
Perineural invasion	Absent	33 (47.8)	1	0.829		
	Present	10 (71.4)	2.53 (−20.97–26.03)			
Extrathyroidal extension	Absent	29 (43.3)	1	0.123	1	0.082
	Present	14 (87.5)	15.74 (−4.46–35.94)		18.05 (−2.37–38.48)	
Lymph node metastasis	Absent	4 (33.3)	1	0.530		
	Present	29 (74.4)	11.12 (−24.6–46.85)			
Extranodal extension	Absent	18 (58.1)	1	0.558		
	Present	14 (73.7)	6.86 (−16.75–30.47)			

## Discussion

Papillary thyroid cancer (PTC) is the most common form of thyroid cancer. It is also rapidly increasing in incidence worldwide. This cancer has quite a good prognosis, with less than 2% mortality at 5 years. However, more than 25% of PTC patients suffer a recurrence in the long term (15).

Rearrangements of *BRAF*, *RAS*, *P53* and *RET* are all strongly associated with thyroid cancer. For *RET* rearrangements, *RET*::*PTC*s are the most frequent molecular alterations reported in PTC (2–5). Several fusion partner genes of *RET* in such rearrangements have been reported, but the major ones are *CCDC6*and *NCOA4* (accounting for more than 90%) (6).

We found that the prevalence of *CCDC6*::*RET*, *NCOA4*::*RET* rearrangements and co-occurrence of both in Thailand were 62.65%, 51.81%, and 30.12%, respectively. These rearrangements were not found in normal thyroid, or in benign tumors. Similarly, high frequencies of *RET*::*PTC* rearrangements have been reported from Taiwan (55%), New Caledonia (70%) and Australia (85%) (16, 17). On the other hand, studies from Korea (6.5%) and Japan (30%) showed low frequencies of *RET*::*PTC* rearrangements in PTC (18, 19). This heterogeneity in *RET*::*PTC* rearrangements may be due to ethnicity, different geographical location and/or differences in environmental exposure. The literature has reported that frequency of *RET* rearrangements in papillary carcinomas ranges from 0% to 87% (20, 21). These differences are partly explained by pre-selection of the analyzed cohort, geographical diversity and different sensitivities of the assays used for detection (20, 22). Naito et al. (23) reported that the prevalence of *RAS* gene mutations in Thai PTC patients was higher than in Japan. Being affected the RET rearrangements according to RET rearrangements/mutations and RAS family mutations are almost always mutually exclusive (23, 24).

This suggests that an existence of a specific environmental agent in northeast Thailand, presumably related to the high level of nitrates in the diet, particularly high salt-preserved fish; Pla-ra, Pla-som and also vegetables, water promotes the progression of thyroid papillary carcinomas (23). Apart from the forementioned majority factors, other putative carcinogens, for example, caffeine, ethanol, hypoxia are able to induce DNA double-strand breaks and generate RET/PTC rearrangements (26–28). As reported previously, the frequency of *CCDC6*::*RET* in sporadic thyroid cancer is higher than that of *NCOA4*::*RET* (8, 9). Moreover, our study revealed no major RET rearrangements in benign tumors, similar to previous studies (13, 21).

*CCDC6*::*RET* rearrangement was significantly associated with the classic histological subtype (41/57) (*p* = 0.035), a result in agreement with previous studies (8–10, 29). Similarly, Guerra et al (4) reported that *CCDC6*::*RET* was the dominant type within sporadic carcinomas and was strongly related to the classic subtype (4). *NCOA4*::*RET* rearrangement was significantly associated with the tall cell subtype (13/16) (*p* = 0.019). In term of clinicopathological markers, the *NCOA4*::*RET* rearrangement was significantly associated with angioinvasion (*p* = 0.001), lymphatic invasion (*p* = 0.003), extrathyroidal extension (*p* = 0.001), lymph node metastasis (*p* < 0.001) and extranodal extension (*p* = 0.013), respectively. This is in agreement with Sugg et al (12) and Mochizuki et al (30) who reported that *NCOA4*::*RET* may cause de-differentiation and more aggressive behavior in papillary thyroid carcinoma (12, 30). Certainly, *NCOA4*::*RET* correlates with a more aggressive phenotype and a more advanced stage of PTC (8, 9, 11–13). Galuppini et al (31), Khan et al (18) and Rogounovitch et al (32) reported that an *NCOA4*::*RET* rearrangement was associated with lymph node metastasis (18, 31, 32).

Univariate analysis revealed *CCDC6*::*RET* mRNA expression levels in patients with small tumor size (1 < T ≤ 2 cm) and the classic subtype was not significant that in contrast to with Rhoden et al. (21). Moreover, the other clinicopathological markers, such as extrathyroidal extension or metastases were not correlated with *CCDC6*::RET, in agreement with Tallini et al (33).

High expression levels of *NCOA4*::*RET* were found, but not significant in patients with the tall-cell subtype, extrathyroidal extension, lymph node metastasis and extranodal extension. Our findings are similar to those of from Powell et al (11) that showed the *NCOA4*::*RET* rearrangement was more frequent in tall-cell tumors: its expression in transgenic mice generated solid tumor subtypes of thyroid cancer with more aggressive and metastatic behavior (11). Martínez et al (34) reported that high detection or expression of *BRAFV600E* and low expression or absence of that *CCDC6*::*RET* rearrangement were associated with extrathyroidal extension. In contrast, a high activity of *RET* (presence of *CCDC6*::*RET* rearrangement or high expression) and low activity of *BRAF* (absence of *BRAFV600E* or its low expression) were associated with lymph node metastasis. It has been suggested that *BRAFV600E* and the *CCDC6*::*RET* rearrangement can predict aggressive PTC (34). The literature suggests that *RET*::*PTC* rearrangements, and especially *NCOA4*::*RET*, can be induced by radiation (23, 35). In such cases, Nikiforov et al (8) and Rabes HM et al (9) reported that in radiation-induced PTC, *NCOA4*::*RET* has an equal or even higher prevalence than *CCDC6*::*RET* fusions (8, 9). Radiation exposure is often associated with *NCOA4*::*RET* and is more common in children 4 years of age or younger. In addition, the latest WHO classification 2022 emphasizes that the telomerase reverse transcriptase (*TERT*) gene mutation in PTC patients correlates with an increased risk of distant metastases and poor prognosis. Moreover, the gain of chromosome 1q in the *PLEKHS1* promoter (*PLEKSH1*p) may cause a poorer outcome in PTCs (36). Therefore, further investigation is required into more data on history of recurrence, radiation history, gene aberration, tumor focality among other aspects to enhance our understanding of *RET*::*PTC* rearrangements.

In our study, tumor tissues from 83 PTC patients were investigated to determine the prevalence of *CCDC6*::*RET* and *NCOA4*::*RET* rearrangements in Thailand and to explore the association between the mRNA expression of *CCDC6*::*RET* and *NCOA4*::*RET* rearrangements and clinicopathology. The *CCDC6*::*RET* rearrangement was significantly associated with less aggressive histological subtypes and absence of certain clinicopathological markers. Moreover, the absence of extrathyroidal and extranodal extension were independent predictive factors for *CCDC6*::*RET*.

In contrast, large tumor size, more aggressive types like the tall-cell type, angioinvasion, perineural invasion or extrathyroidal extension were independent predictive factors of *NCOA4*::*RET*. Briefly, *CCDC6*::*RET* correlated with less aggressive, but *NCOA4*::*RET* correlated with more aggressive phenotypes of PTC. In conclusion, the two rearrangements that we studied were strongly associated with particular clinicopathological phenotypes, particularly histological subtype.

## Data Availability

The datasets presented in this study can be found in online repositories. The names of the repository/repositories and accession number(s) can be found in the article/Supplementary Material.
