# Supplementary material for: RET rearrangements are relevant to histopathologic subtypes and clinicopathological features in Thai papillary thyroid carcinoma patients
Source: Pathol Oncol Res. 2023 Apr 28;29:1611138. doi: 10.3389/pore.2023.1611138 (PMC10175595; doi:10.3389/pore.2023.1611138)
Supplement: Supplementary file 1 [file Table1.pdf]

Table S1: RT-PCR conditions for *CCDC6::RET* and *NCOA4::RET* detection

CCDC6::RET	**Temperature (˚C)**	**Time**	**Cycle(s)**
Hold stage	95	4 mins	1
PCR stage	95	30s	40
	50	30s	
	72	30s	
Final extension stage	72	7 mins	1
Melt curve stage	95	15s	1
	52	1 min	
	72	15s	
NCOA4::RET	**Temperature (˚C)**	**Time**	**Cycle(s)**
Hold stage	95	4 mins	1
PCR stage	95	30s	40
	52	30s	
	72	30s	
Final extension stage	72	7 mins	1
Melt curve stage	95	15s	1
	52	1 min	
	72	15s	
